# Oncologic outcomes comparison of partial ureterectomy and radical nephroureterectomy for urothelial carcinoma

**DOI:** 10.1186/s12894-019-0557-2

**Published:** 2019-11-21

**Authors:** Shengxian Li, Yuchen Pan, Jinghai Hu

**Affiliations:** 1 Department of Urology, First Hospital of Jilin University, Changchun, 130021 People’s Republic of China; 2 Key Laboratory of Polymer Ecomaterials, Changchun Institute of Apllied Chemistry, Chinese Academy of Sciences, Changchun, 130022 People’s Republic of China; 3 Department of Clinical Epidemiology, First Hospital of Jilin University, Changchun, 130021 People’s Republic of China

**Keywords:** Urothelial carcinoma, Partial ureterectomy, Radical nephroureterectomy, Caner specific survival

## Abstract

**Background:**

The appropriate application of various treatment for upper tract urothelial carcinomas (UTUCs) is the key to prolong the survival of UTUC patients. Herein, we used data in our database to assess the oncological outcomes between partial ureterectomy (PU) and radical nephroureterectomy (RNU).

**Methods:**

From 2007 to 2014, 255 patients with UTUC undergoing PU or RNU in our hospital database were investigated. Perioperative, postoperative data, and pathologic outcomes were obtained from our database. Cancer-specific survival (CSS) was assessed through the Kaplan-Meier method with Cox regression models to test the effect of these two surgery types.

**Results:**

The mean length of follow-up was 35.8 months (interquartile range 10–47 months). Patients with high pT stage (pT2–4) suffered shorter survival span (HR: 9.370, 95% CI: 2.956–29.697, *P* < 0.001). There were no significant differences in CSS between PU and RNU (*P* = 0.964). In the sub-analysis, CSS for RNU and PU showed no significant difference for pTa–1 or pT2–4 tumor patients (*P* = 0.516, *P* = 0.475, respectively).

**Conclusions:**

PU is not inferior to RNU in oncologic outcomes. Furthermore, PU is generally recognized with less invasive and better renal function preservation compared with RNU. Thus, PU would be rational for specific patients with UTUCs.

## Background

Upper tract urothelial carcinomas (UTUCs), as a kind of urothelial carcinomas (UCs), accounting for only 5–10% [1]. Generally, 60% of UTUCs are invasive [2]. Seventy to Ninety years old showed the highest morbid risk among all age groups and are three times more ordinary in men [2]. UTUC with pure nonurothelial histology is quite rare, but about 25% of cases have variants. Computed tomography (CT) is an available imaging technique with high diagnostic accuracy [3]. In about 90% of cases, the tumor grade can be determined with a low false negative rate [4]. Ureteroscopic biopsy combining with urinary cytology, and imaging findings such as hydronephrosis may contribute for the decision of surgical type like partial ureterectomy (PU) and radical nephroureterectomy (RNU) [5]. Tumor stage and grade, lymph node involvement, lymphovascular invasion, surgical margins, and pathological factors could be used to estimate prognosis [2]. Nowadays, the concept for preservation of renal function has been emphasized. For low-risk UTUC, kidney-sparing surgery (KSS) unlike radical surgery could preserve kidney function without compromising oncological outcomes. KSS such as PU are performed for the patients with solitary kidney, renal insufficiency, or low-risk tumors. RNU is still the standard treatment for high-risk UTUCs, no matter where the tumor location is [2, 6]. Herein, we used data in our database to assess the oncological outcomes between PU and RNU.

## Methods

### Study population and treatment

The research protocol was approved by the ethics committee of Jilin University’s Institutional Ethical Review Board, and written informed consent was obtained. Three hundred thirty two patients with UTUC from January 2007 to December 2014 in our database were included in First Hospital of Jilin University. Of them, 52 patients were excluded for suspected multifocal tumors in the upper urinary tract preoperatively. Another 25 patients were excluded for distant metastases (*n* = 14), no surgical treatment (*n* = 3), suffering previous or concurrent radical cystectomy (*n* = 8). Among the remaining 255 patients, 182 of them underwent RUN, 73 of them underwent PU. All Patients were evaluated disease progression by CT preoperatively. During the study period, ureteroscopy with biopsy was not routinely performed. The choice and surgical technique of RNU and PU could not be standardized. Surgeon preferences have a significant impact on the selection and surgical technique of RNU and PU. For RNU, the kidney and entire ureter with a bladder cuff were excised. PU was performed along with the excision of ureteral segment. There were no statistically differences between age at surgery, sex, tumor side, presence of hematuria, and presence of hydronephrosis. Clinical and pathological staging was in accordance with TNM classification 2002 [7, 8].

### Statistical analysis

Outcomes were measured by survival time (from the time of surgery to cancer specific mortality). Continuous variables were described as the median (interquartile range). T tests or nonparametric tests were carried out to assess the differences in variables with a continuous distribution. Categorical variables were described as the frequency (percentage) and analyzed by the Chi-square or Fisher exact test. The Cox proportional hazard models were employed for univariate and multivariate analyses to determine whether surgery type (RNU vs PU) and other variables was associated with CSS. The survival curves of each stratified variablewere plotted by the Kaplan-Meier method and compared by log-rank test. SPSS version 18.0 (SPSS Inc., Chicago, IL) was used for statistical analyses. All *P* values were two-tailed, and P value < 0.05 indicated statistical significance.

## Results

### Clinicopathological characteristics

The mean length of follow-up was 35.8 months (interquartile range 10–47 months). The baseline characteristics of the patients are shown in Table 1. The mean age and percent of male in RNU group were 66.74 ± 8.41 and 49.5%, respectively; 67.62 ± 10.09 and 41.4% in PU group, respectively. Smokers in RNU group were much more than in PU group (38.2% vs 15.6%, *P* = 0.021). In RNU group, half of the patients had tumor in the left kidney (54.1%). PU group is contrary to it (39.7%). The proportion of patients with hydronephrosis, hematuria or abnormal renal function in RNU group were close to PU group (*P* > 0.05). Compared with PU group,operation time of patients in RNU group was much longer (157.50 (116.25–180.00) vs 95.00 (76.25–126.25), *P* < 0.001). In RNU group, nearly half (42.0%) were diagnosed at pT stage1, 5.2% at pT stage a, 27.6% at pT stage 2, 23.6% at pT stage 3, and only 1.7% at pT stage4. In PU group, the proportion is 49.3, 3, 37.3, 9, and 1.5%, respectively.

**Table 1:** Clinical and pathologic characteristics of the two groups

Variables	RNU	PU	t/χ^2^	*P*
Age	66.74 ± 8.41	67.62 ± 10.09	−0.709	0.479
Sex				
Male	90 (49.5%)	30 (41.4%)	1.460	0.227
Female	92 (50.5%)	43 (58.9%)		
Smoking				
No	47 (61.8%)	27 (84.4%)	5.300	0.021
Yes	29 (38.2%)	5 (15.6%)		
Tumor side				
Left	98 (54.1%)	29 (39.7%)	4.325	0.038
Right	83 (45.9%)	44 (60.3%)		
Hydronephrosis				
No	18 (10.3%)	11 (16.4%)	1.727	0.189
Yes	157 (89.7%)	56 (83.6%)		
Hematuria				
No	59 (33.7%)	30 (44.1%)	2.283	0.131
Yes	116 (66.3%)	38 (55.9%)		
Renal function				
Normal	48 (43.6%)	20 (47.6%)	0.195	0.659
Abnormal	63 (56.4%)	22 (52.4%)		
Operation time	157.50 (116.25–180.00)	95.00 (76.25–126.25)	−4.476	< 0.001
pT stage			8.287	0.067
pTa	9 (5.2%)	2 (3.0%)		
pT 1	73 (42.0%)	33 (49.3%)		
pT 2	48 (27.6%)	25 (37.3%)		
pT 3	41 (23.6%)	6 (9.0%)		
pT 4	3 (1.7%)	1 (1.5%)		
Lymphovascular invasion	4 (2.2%)	0 (0%)	1.630	0.202

*RUN* Radical nephroureterectomy, *PU* Partial ureterectomy

### Oncological outcome

**Fig. 1:**
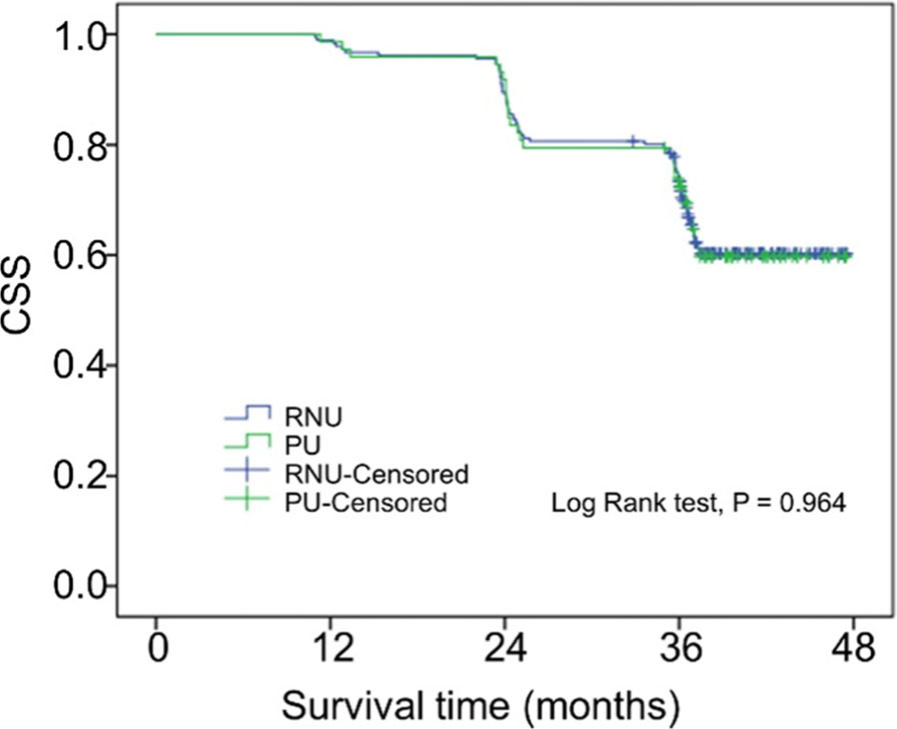
Kaplan–Meier estimates of CSS according to surgery type in patients with UTUC

**Fig. 2:**
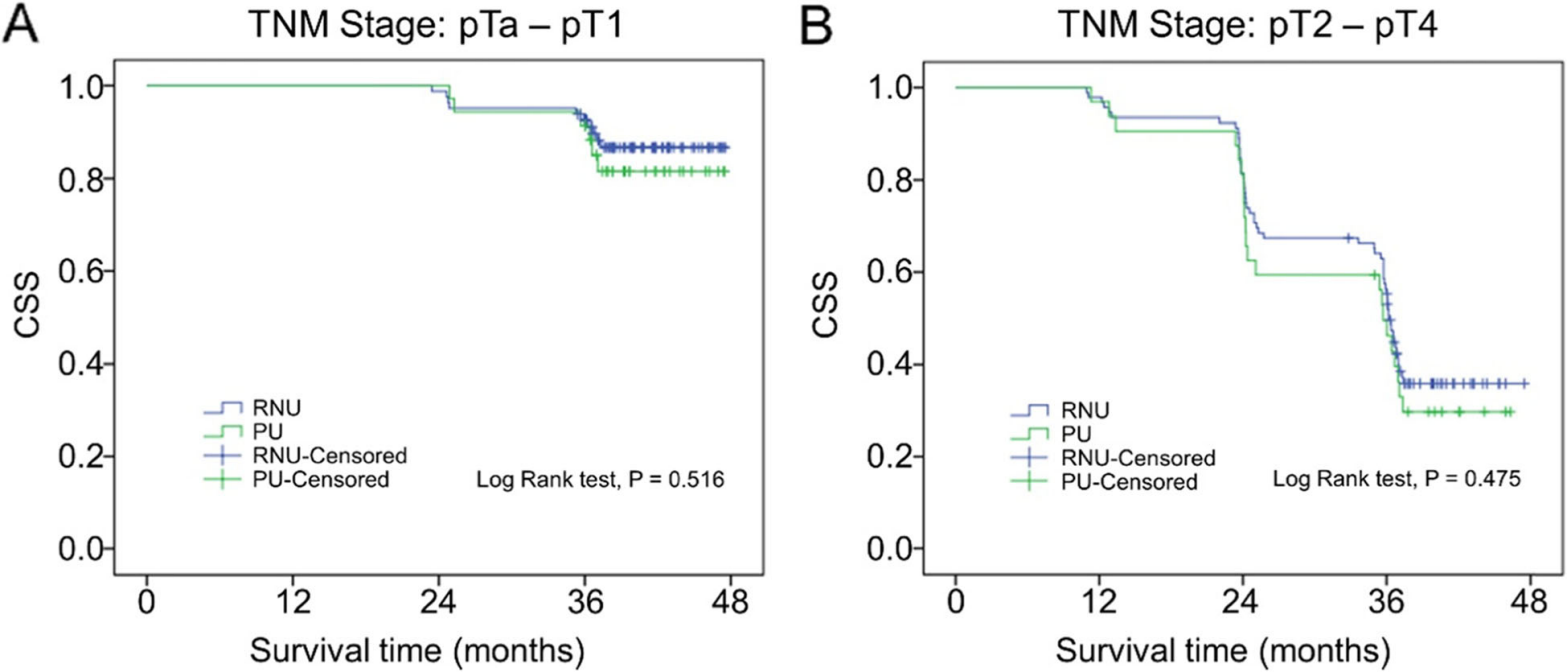
Kaplan–Meier estimates of CSS according to surgery type in patients with pTa – pT2 (**a**) and pT2 – pT4 (**b**) UTUC

Univariable and multivariable analyses for CSS after surgery were shown in Table 2. On multivariable analysis, pT stage was significantly associated with CSS. The results showed that patients with high pT stage (pT2–4) suffered shorter survival span (HR: 9.370, 95% CI: 2.956–29.697, *P* < 0.001). The independent predictors of CSS were higher pT (*P* < 0.001; Table 2). The specific results are exhibited in Table 2. Furthermore, there were no significant differences in CSS between PU and RNU (*P* = 0.964) (Fig. 1). In the sub-analysis, there was no significant difference in 3-year CSS probability between pTa–1 tumor patients treated with RNU (87.7%) and PU (82.9%) (*P* = 0.516). Similarly, for patients with pT2–4 tumors, 3-year CSS for RNU and PU showed no significant difference (38.0% vs 31.3%, *P* = 0.475, respectively) (Fig. 2).

**Table 2:** Cox regression analysis of factors associated with CSS

Variables	Univariate			Multivariate		
	HR	95% CI	*P*	HR	95% CI	*P*
Age (years)						
≤ 70	1.000			1.000		
> 70	0.885	0.581–1.348	0.569	1.240	0.488–3.150	0.652
Sex						
Male	1.000			1.000		
Female	0.713	0.478–1.062	0.096	1.328	0.477–3.693	0.587
Smoking						
No	1.000			1.000		
Yes	1.095	0.548–2.190	0.797	0.695	0.225–2.147	0.527
Tumor side						
Left	1.000			1.000		
Right	0.726	0.486–1.084	0.117	1.136	0.451–2.861	0.787
Hydronephrosis						
No	1.000			1.000		
Yes	1.134	0.588–2.188	0.707	0.742	0.149–3.698	0.716
Hematuria						
No	1.000			1.000		
Yes	1.307	0.842–2.029	0.233	2.405	0.820–7.048	0.110
Renal function						
Normal	1.000			1.000		
Abnormal	1.130	0.688–1.911	0.650	1.503	0.613–3.686	0.374
pT stage						
pTa–1	1.000			1.000		
pT2–4	6.907	4.028–11.845	< 0.001	9.370	2.956–29.697	< 0.001
Surgical intervention						
RNU	1.000			1.000		
PU	1.010	0.651–1.567	0.964	1.705	0.608–4.778	0.310

*RUN* Radical nephroureterectomy, *PU* Partial ureterectomy

## Discussion

UTUC is a kind of rare malignancy. The main treatments for URUC are RNU and KSS like PU. Recent data indicated that patients with low grade UTUC could be treated by PU effectively. The guidelines of the European Association of Urology suggested that PU is a legitimate therapy for small, unifocal, low grade UTUC with no signs of infiltration [2, 7, 9], and indicated that RNU is still the standard treatment for high-risk UTUCs [2, 10]. However, Bagrodia et al. compared the prognosis of UTUC patients who suffered either RNU or PU. They suggested that PU seemed to possess oncologic efficacy equal to RNU. Meanwhile, PU could preserve the renal function to the maximum extent [9]. Similar results were also demonstrated in the investigation from Fukushima et al. [11]. Colin et al. conducted a multicenter cohort study of UTUC which suggested that surgical interventions was not the independent prognostic factor for recurrence-free survival (RFS) and CSS [6]. The investigation from Hung et al. indicated that the local recurrences, bladder recurrences, distant metastasis, and CSS showed no significant differences between RNU and PU [12]. These findings mentioned above would show the ability of PU to maximize the preservation of renal function in carefully selected patients.

In the current study, we noticed that pathological stage remained the most important predictor for the CSS of patients with UTUCs. The results also revealed that CSS showed no significant differences between RNU and PU in both low pT stage (pTa–1) and high pT stage (pT2–4) UTUCs. The probable reason is that the number of patients involved in our study is not large enough, thus this study may not be powerful enough to show significance [6]. Furthermore, there were some selection biases that RNU group seemed to have more aggressive pathological features compared with PU group, because there was not the standardized indication for PU and surgeon preferred PU for lower stage UTUCs [13, 14]. Selection biases can lead to better survival in PU group compared with RNU group, though the difference was not significant in our analysis. Our findings, and similar findings from other researchers, indicated that PU could act as a selectable treatment for selected UTUC patients [15]. In addition, pathologists with expertise rechecked all the pathologic specimens, and all pathologic results were collected standardly.

This study is retrospective study, and the number of involved patients is relatively small. These two characteristics are the main limitations of this study. Because a prospective randomized controlled study of PU compared with RNU is difficult due to the rarity of UTUC. Secondly, we could not ascertain whether PU was done imperatively or electively. Furthermore, surgeon preferences have a great impact on the surgical technique and indication for RNU and PU, which could not be determined standardly. Nor can we assess which cases are suitable for endoscopic treatment when receiving RNU therapy [16, 17].. Multiple surgeons were employed to operate on the investigated patients. This situation might lead to a selection bias and variable surgical techniques. Finally, another limitation of our research is that we could not provide the information about tumor grade and metastases due to the sample problem.

## Conclusion

In conclusion, PU and RNU might possess equivalent long-term oncologic outcomes. Furthermore, PU could reduce the adverse outcomes of nephron loss caused by RNU. PU could be rational selection for selected patients with UTUCs. Further randomized researches compared with PU and RNU are still necessary to support the results. Prospective trials with large sample sizes are essential to more accurately assess the role of PU in UTUC.

## Data Availability

The data that support the findings of this study are available from the corresponding author on reasonable request.

## Abbreviations

CSS: Cancer-specific survival
CT: Computed tomography
KSS: Kidney-sparing surgery
PU: Partial ureterectomy
RFS: Recurrence-free survival
RNU: Radical nephroureterectomy
UC: Urothelial carcinomas
UTUC: Upper tract urothelial carcinoma
